# Supplementary material for: He Karanga Maha. Investigating Relational Resource Management in Aotearoa, New Zealand
Source: Int J Environ Res Public Health. 2023 Apr 18;20(8):5556. doi: 10.3390/ijerph20085556 (PMC10138379; doi:10.3390/ijerph20085556)
Supplement: Supplementary file 1 [file ijerph-20-05556-s001.zip › ijerph-2212595-supplementary.pdf]

**Table S1.** Glossary of Māori language terms.

| Reference | Term | Definition |
|---|---|---|
| [50] | Māori | Indigenous person of Aotearoa/New Zealand |
| [6] | Whakapapa | The genealogical ties that bind people with people, with the environment, and with the cosmos |
| [15] | Kaitiakitanga | Guardianship and resource management |
| [15] | Kaitiaki | An agent of kaitiakitanga |
| [50] | Iwi | Nation descended from a common ancestor and associated with a distinct territory |
| [50] | Hāpu | Kinship group and the primary political unit in traditional Māori society. A number of related hapū usually shared adjacent territories forming an iwi. |
| [50] | Whānau | Extended family, family group, a familiar term of address to a number of people. |
| [50] | Mana whenua | Territorial rights, power from the land, authority over land or territory, jurisdiction over land or territory - power associated with possession and occupation of tribal land. |
| [11] | Mana | Spiritual authority |
| [11] | Tapu | Sacredness, set apart |
| [11] | Manaaki | Hospitality |
| [11] | Tuku | Transfer, gift, release |
| [18] | Kaupapa Māori | Kaupapa Māori research methodologies have arisen out of mātauranga Māori [Māori knowledge] as a theory and analysis of the approaches to research which involve Māori. It legitimises Māori epistemology and is positioned to address the concerns of Māori. |
| [50] | Ōi | Grey-faced petrel, *Pterodroma macroptera*, northern mutton-bird. |
| [50] | Tītī | Muttonbird, sooty shearwater, *Puffinus griseus*, young of the sooty shearwater. |
| [50] | Kaumātua | Adult, elder, elderly man, elderly woman, old man - a person of status within the whānau. |
| [50] | Ngāti Wai | An iwi of the Northland Region of the North Island, New Zealand |
| [50] | Te Kawerau ā Maki | An iwi of the Auckland region in the North Island, New Zealand |
| [50] | Whanaungatanga | Relationship, kinship, sense of family connection - a relationship through shared experiences and working together which provides people with a sense of belonging. |
| [50] | Whangārei | A city in the North Island of New Zealand |
| [50] | Taiharuru | An offshore island in the North Island of New Zealand. |
| [50] | Karakia | Incantation, ritual chant, chant, intoned incantation, charm, spell - a set form of words to state or make effective a ritual activity. |
| [50] | Pātaua | A settlement in the Northland Region of New Zealand |
| [50] | Te Henga | Bethells Beach, Auckland, New Zeakand |

| | | |
|---|---|---|
| [50] | Harakeke | New Zealand flax, *Phormium tenax* |
| [50] | Kete | Basket, kit |
| [50] | Wairua | Spirit, soul |
| [50] | Taonga | Treasure, anything prized - applied to anything considered to be of value including socially or culturally valuable objects, resources, phenomenon, ideas, and techniques. |
| [50] | Whenua | Land |
| [50] | Māra | Garden, cultivation |
| [50] | Kai | Food, meal |
| [50] | Taukihepa | Big South Cape Island |
| [50] | Rakiura | Stewart Island |
| [50] | Kōrero | Speech, narrative, story, news, account, discussion, conversation, discourse, statement, information |
| [50] | Ihumoana | An offshore island at Bethells Beach |
| [50] | Kaimoana | Seafood or shellfish |
| [50] | Kauwahaia | An offshore Island at O'Neil's Beach. |
| [50] | Pā (site) | Ancestral burial grounds |
| [50] | Rāhui | A temporary ritual prohibition, closed season, ban, reserve. |
| [50] | Tikanga | Correct procedure, custom, habit, lore, practice, protocol |
| [50] | Tangata whenua | Local people, hosts, indigenous people - people born of the whenua |
| [50] | Tūpuna | Ancestors, grandparents |
| [50] | Mokopuna | Grandchildren, grandchild, descendants. |
| [50] | Tangata/Tāngata | Person, man, human being, individual |
| [50] | Mauri | Life principle, life force, vital essence, special nature, a material symbol of a life principle, source of emotions - the essential quality and vitality of a being or entity |
| [50] | Tupua | Strange, supernatural, abnormal |
| [50] | Atua | Ancestor with continuing influence, god, demon, supernatural being, deity. |
| [50] | Māui | An ancestor of Polynesia |
| [50] | Tohunga | Skilled person, chosen expert, priest, healer |
| [50] | Taniwha | Water spirit, monster, dangerous water creature, powerful creature, chief, powerful leader, something or someone awesome - taniwha take many forms from logs to reptiles and whales and often live in lakes, rivers or the sea. They are often regarded as guardians by the people who live in their territory, but may also have a malign influence on human beings |
| [50] | Lake Wainamu | A Lake in West Auckland, New Zealand. |
| [50] | Waitākere River | A River in West Auckland, New Zealand |
| [50] | Mahi | Work, job, employment, trade (work), practice, occupation, activity, exercise, operation, function |
| [50] | Mana | Prestige, authority, control, power, influence, status, spiritual power, charisma - mana is a supernatural force in a person, place or object |

| | | |
|---|---|---|
| [50] | Manaakitanga | Hospitality, kindness, generosity, support - the process of showing respect, generosity and care for others |
| [50] | Kuia | Elderly woman, grandmother, female elder |
| [50] | Ihumatao | A Region of South Auckland, New Zealand |
| [50] | Õkāhu Bay | A bay in Auckland, New Zealand |
| [50] | Pipi | *Paphies australis* - a common edible bivalve with a smooth shell found at low tide just below the surface of sandy harbour flats. Hinge is near the middle of the shell |
| [50] | Māngere | A suburb in the city of Auckland, in the North Island of New Zealand |
| [50] | Pūpū | Common cat's eye, cat's eye turban shell, *Turbo smaragdus* |
| [50] | Taupo | A town in the central North Island of New Zealand |
| [50] | Reo | Language, dialect, tongue, speech |
| [50] | Pākehā | New Zealander of European descent - probably originally applied to English-speaking Europeans living in Aotearoa/New Zealand |
